# Executive Subdomains Are Differentially Associated With Psychosocial Outcomes in Major Depressive Disorder

**DOI:** 10.3389/fpsyt.2018.00309

**Published:** 2018-07-10

**Authors:** Matthew J. Knight, Bernhard T. Baune

**Affiliations:** Discipline of Psychiatry, Adelaide Medical School, University of Adelaide, Adelaide, SA, Australia

**Keywords:** depression, MDD, cognition, executive functioning, forward planning, updating, inhibition, psychosocial functioning

## Abstract

**Background:** Deficits in executive functioning are frequently associated with poor psychosocial outcomes in Major Depressive Disorder (MDD). However, there is a poor understanding of the domain-specific relationships between executive subdomains (e.g., forward planning, decision making) and specific psychosocial issues (e.g., occupational functioning, social relationships). The current study explored these relationships across currently depressed and remitted MDD patients, as well as a healthy control group.

**Methods:** Data from 142 participants were obtained from the Cognitive Functioning and Mood Study (CoFaM-S), a cross sectional study of mood, cognition, and psychosocial functioning in mood disorders. Participants' [current depression *n* = 31, remitted depression *n* = 52, healthy controls (HC) *n* = 59] executive functioning was evaluated with well-established tests of executive subdomains (i.e., Tower of London, card sorting, Stroop task). The Functioning Assessment Short Test (FAST) was employed to clinically evaluate psychosocial dysfunction.

**Results:** The results indicated that forward planning was most strongly associated with psychosocial issues in the current depression group as compared to HCs, while cognitive updating was primary in the remitted group vs. HC.

**Conclusions:** These findings suggest that executive subdomains are deferentially associated with psychosocial issues across different stages of depressive illness, and that forward planning and cognitive updating should be considered in adjunctive cognitive treatment.

## Introduction

While Major Depression is characterized by negative mood, cognitive dysfunction is increasingly recognized as a primary symptom (1, 2). The profile of cognitive domains affected by MDD is yet to be fully explored, however recent evidence suggests that deficits in executive functioning, attention, working memory, processing speed, and verbal/linguistic fluency are common (3–6) and present in acute as well as remitted depression (7–9). Cognitive deficits are frequently maintained despite improvement and remittance of mood symptoms (1, 10), highlighting their independence to other symptoms of MDD, and the resistance of cognitive deficits to current treatment approaches (e.g., CBT, SSRIs). Mounting evidence suggests that cognitive deficits contribute independently to dysfunction in the daily lives of individuals with MDD (4, 11). In particular, cognitive deficits are implicated in the pathology of psychosocial dysfunction in domains of occupational functioning, daily life, social relationships, and self-perceived quality of life (4, 11, 12). The broad relationship between cognitive impairment and psychosocial functioning underscores the importance of research in this area. Specifically, there is a clear need to determine which domains of cognition most are most strongly associated with psychosocial outcomes, and hence which domains are appropriate treatment targets for adjunctive and developing therapies (13, 14). This significant gap in our understanding is addressed by the current research (15), which investigates the role of specific cognitive issues in functional disability in MDD.

A review of the relationship between cognition and psychosocial functioning in MDD identified that executive functioning plays an important role in several psychosocial outcomes (e.g., occupational productivity, daily responsibilities) (4). One study identified that executive dysfunction predicted longitudinal clinical and functional prognosis more strongly than the original psychiatric diagnosis, while another found that executive dysfunction mediated the relationship between depression and impaired daily responsibilities (16). In addition, research by Knight et al. (17) in remitted depression suggests that executive deficits are associated with psychosocial dysfunction more strongly than other cognitive domains (e.g., attention, semantic fluency). Taken together, current literature highlights the clinical significance of executive functioning in its independent contribution to functional outcomes and long-term recovery (7, 10).

It is worth considering why executive functioning contributes more broadly to functional outcomes in MDD in comparison to other cognitive domains (e.g., visuospatial processing, immediate memory). A plausible explanation is that executive functioning is applied in a wide range of cognitive and behavioral executive subdomains, which in turn are necessary to maintain adaptive psychosocial functioning. These executive subdomains include decision making (18), set-shifting (15), forward planning (19), problem solving (8, 19), working memory (1, 20), cognitive flexibility (21), cognitive updating (15, 22), and inhibition (5, 23). It follows that deficits in executive functioning are likely to be detrimental across a number of executive subdomains, which in turn may negatively interact with a broad range of psychosocial domains.

Some evidence for the mechanism of executive subdomains in psychosocial outcomes comes from research in the domain of occupational functioning (5). Godard et al. conducted a 12-month prospective study with unipolar depression patients, in which clinical, cognitive, and psychosocial outcomes were assessed at baseline and over a 12-month follow-up period. The results indicated that executive dysfunction in subdomains of inhibition, cognitive flexibility, and updating was prevalent at baseline and was maintained at 1-year follow-up. In addition, executive dysfunction was associated with losses in occupationally functioning, with participants reporting decreased ability to resolve problems, develop strategies, and manage simultaneous tasks. These results provide support for the notion that executive deficits transfer to a broad range of executive subdomains, which in turn negatively interact with psychosocial outcomes.

The aim of the current study was to evaluate the domain-specific relationships between executive subdomains (e.g., updating, inhibition) and psychosocial functioning. It was expected that dysfunction in executive subdomains would be associated with psychosocial disability, however based on the literature, it could not be hypothesized which executive subdomains would be related to specific psychosocial deficits (e.g., autonomy, occupational functioning). Cognitive and psychosocial disability was assessed in currently depressed and remitted depressed patients, as well as a healthy control group, as the cognition-functioning relationship may differ between these groups (4, 7, 17).

## Materials and methods

Cross-sectional data were analyzed from the Cognitive Function and Mood study (CoFaM-S) (24), a multi-site investigation of cognitive, social, emotional, and functional status in patients with mood disorders. The CoFaM-S study was reviewed by the Human Research Ethics Councils (HRECs) of the Royal Adelaide Hospital (approval number: 111230) and the University of Adelaide (approval number: H-160-2011), and was conducted in accordance with the Declaration of Helsinki. All participants were verbally informed about study procedures, and read a study information sheet, before providing written consent to participate. CoFaM-S participants were at least 18 years of age, and no age limit was imposed. Inclusion criteria included lifetime diagnosis of a mood disorder [unless entered as a healthy control (HC)] according to DSM-IV-TR criteria (25). Participants were recruited from inpatient and outpatient clinics throughout Eastern, Western, and Northern Mental Health Networks in Adelaide, South Australia, as well as through advertisement in the general community. Exclusion criteria included diagnosis of psychotic disorders, dementia, learning disorders, eating disorders, autism spectrum disorder, or medical illnesses which could affect cognitive functioning (e.g., multiple sclerosis) (24). The MINI 600 Neuropsychiatric Diagnostic Interview was used to screen patients for psychiatric illness (26), and the Hamilton Depression Scale (HAM-D) measured symptom severity (27).

Participants (*N* = 142) were in included in the current study on the basis of completing measures of executive cognition and psychosocial functioning. Individuals were defined as currently depressed (*N* = 31) if primary symptoms of MDD (i.e., prolonged negative mood, lack of interest) were experienced over the past 2 weeks as reported by the MINI (27). Inclusion criteria for currently depressed patients permitted secondary symptoms associated both MDD and other illnesses (e.g., anxiety symptoms), however depressed mood was considered primary according to the results of the MINI. The remitted group (*N* = 52) was composed of individuals who reported a history of MDD, but were free of MDD symptoms in the past 2 weeks according to MINI criteria. Remitted participants also demonstrated HAM-D scores <7, indicating “normal” mood (27, 28). Remitted and healthy groups demonstrated anxiety symptoms within the “normal” range, while currently depressed subjects indicated “mild” anxiety symptoms^1^ (29). Average duration of illness in the current and remitted depression groups was 13 years (*SD* = 11.09). Mean number of previous episodes of depression was very similar in the current (*M* = 1.97, *SD* = 1.45) and remitted (*M* = 1.27, *SD* = 1.44) depression groups. Healthy individuals (*N* = 59) included those who reported no lifetime presence of MDD or any other psychiatric illness included in the MINI 600. Five currently depressed and 16 remitted participants were taking antidepressant medication. The mean age of participants was 32.6 (*SD* = 15.73), 62% (88) were female, and mean years of education was 13.41 (*SD* = 2.26). One-way ANOVAs and a chi-squared test indicated that mean age, years of education, and sex did not differ between the currently depressed, remitted, and healthy groups (all *p*s > 0.05). Additional demographic and psychiatric information stratified by MDD status (current depression, remitted depression, healthy) is presented in Table 1.

**Table 1:** Demographic characteristics by MDD status (total, current depression, remitted depression, healthy).

	**Total sample (*N* = 142)**	**Current depression (*n* = 31)**	**Remitted depression (*n* = 52)**	**Healthy (*n* = 59)**
Gender				
Female	*n* = 88 (62%)	*n* = 21 (67%)	*n* = 32 (63%)	*n* = 34 (58%)
Male	*n* = 54 (38%)	*n* = 10 (33%)	*n* = 20 (37%)	*n* = 25 (42%)
Age	*M* = 32.58 (*SD* = 15.73)	*M* = 35.30 (*SD* = 16.17)	*M* = 33.7 4 (*SD* = 16.56)	*M* = 30.23 (*SD* = 14.79)
Years of education	*M* = 13.41 (*SD* = 2.26)	*M* = 12.97 (*SD* = 2.16)	*M* = 13.45 (*SD* = 2.00)	*M* = 13.53 (*SD* = 2.51)
HAM-D score	*M* = 9.20 (*SD* = 3.55)	*M* = 16.11 (*SD* = 6.99)	*M* = 6.66 (*SD* = 5.15)	*M* = 4.82 (*SD* = 5.51)
HAM-A score	*M* = 9.31 (*SD* = 8.20)	*M* = 19.29 (*SD* = 8.24)	*M* = 8.04 (*SD* = 6.01)	*M* = 5.27 (*SD* = 5.12)
Lifetime presence of anxiety disorder	*n* = 44 (31%)	*n* = 19 (61%)	*n* = 25 (48%)	0 (0%)
Lifetime presence of bipolar disorder	*n* = 1 (0.7%)	*n* = 1 (3.2%)	*n* = 0 (0%)	0 (0%)

Executive performance was assessed with several well-established psychological assessments in the Psychology Experiment Building Language (PEBL) software (30) [i.e., the Stroop task, Tower of London (TOL), Berg's Card Sorting Test (BCST)^2^]. Performance in individual tasks was used as an indication of functioning in executive subdomains. Specifically, incongruency errors in the Stroop Task indicated inhibition (23), total moves in the TOL task indicated forward planning (19), perseverative errors in BCST indicated updating (31) (see Supplementary eTable 1). The psychometric properties of the included tests, as well as their construct validity as measures of executive subdomains, are well established in cognitive and clinical literature (8, 21, 31).

Psychosocial functioning was assessed with the Functioning Assessment Short Test (FAST); a clinician rated interview gauging the extent of dysfunction across six functional domains (i.e., autonomy, occupational functioning, cognitive functioning, financial issues, leisure time, and interpersonal relationships). Functional impairment is rated on a 4 point scale, with 0 indicating no dysfunction and 3 indicating severe impairment. Six composite FAST ratings were derived from the mean score of impairment in each functional subdomain (i.e., autonomy (Range: 0–12), occupational dysfunction (Range: 0–15), subjective cognition (Range: 0–15), financial issues (Range: 0–6), interpersonal relationships (Range: 0–18), and leisure time (Range: 0–6). Overall psychosocial functioning (i.e., FAST total score) is indicated by the sum of FAST subdomains (Range: 0–72). FAST subdomain composites and total FAST score were used as outcome variables in separate simultaneous regression analyses.

### Statistical analyses

Simultaneous linear regression analyses were conducted with SPSS for windows, version 24. Performance metrics for executive subdomains (see Supplementary eTable 1) were entered as independent variables. Age, sex, and years of education were entered as covariates, as these factors can affect the relationship between cognition and psychosocial functioning (4, 32). HAM-D total score was entered as a covariate in the current and remitted depression regression models. In the HC group, separate analyses were performed with HAM-D score either included or excluded as a covariate in the HC regression model. This approach was elected because reported symptoms in the HAM-D in HCs may reflect transient issues associated with the time of testing, rather than mental illness. Accordingly, it is pertinent to run separate analyses to determine whether reported symptoms in the HAM-D influenced the relationship between executive subdomains and psychosocial functioning in HCs. Statistics for the HC regression model with HAM-D scores included are reported in Supplementary eTable 3.

The dependent variable in the initial analysis was Total FAST score (i.e., overall psychosocial functioning). In each of the six subsequent regression analyses, a single composite FAST subdomain score (i.e., autonomy, occupational functioning, cognitive functioning, financial issues, leisure time, and interpersonal relationships) was entered as a dependent variable. These analyses were performed separately for the currently depressed, remitted, and HC groups. This model enabled evaluation of the relationship between executive subdomains and specific psychosocial issues using standardized beta coefficients, adjusted for HAM-D score, age, sex, and years of education. Presence of lifetime anxiety was also included as a covariate in initial analyses, however this factor was not associated with total FAST score in the current or remitted depression groups, nor did inclusion of this factor significantly influence the association between executive subdomains and FAST total score. Presence of anxiety was therefore removed from subsequent analyses, as anxiety symptoms are closely associated with depressive symptoms (33) and could therefore reduce the sensitivity of portioning analyses by current and remitted depression.

Multicollinearity of executive subdomain variables was low, as indicated by variance inflation factors <4. *Post-hoc* power analyses were conducted on standardized beta coefficients within the participant groups (i.e., remitted depression, current depression, HCs) with the Gpower software (34). Given the sample sizes of these groups, three independent variables, and the “medium” effect sizes identified (β = 0.215–0.420), analyses in the remitted depression group achieved 99% power to detect a relationship between executive subdomains and FAST outcomes, whereas the currently depressed and HC group indicated 98% power. Descriptive statistics (i.e., means, *SD*s) for FAST total score, FAST subdomains, and executive subdomains are provided for each clinical group (current depression, remitted depression, healthy) in Supplementary eTable 2.

## Results

### Healthy controls

Analysis of total FAST score in the healthy group indicated that no executive subdomain was significantly associated with overall psychosocial functioning after adjusting for age, sex, and years of education. Age (β = 0.312, *p* = 0.011) and years of education (β = −0.354, *p* = 0.008) were associated total FAST score, with older patients demonstrating greater dysfunction, and higher educated patients demonstrating reduced dysfunction. Separate regression analyses employed individual FAST subdomain composite scores as outcome variables. Analyses of autonomy, occupational functioning, subjective cognition, and leisure time mirrored that of total FAST score, finding no statistically reliable relationship between executive subdomains and functional outcomes. In contrast, analysis of interpersonal relationships and financial issues indicated that incongruency errors in the Stroop task (i.e., inhibition) were independently associated with functional outcome, with greater errors associated with increased dysfunction. Beta coefficients for the relationship between executive subdomains and functional outcomes, as well as associated *p*-values, in the healthy group are reported in Table 2,^3^.

**Table 2:** Domain specific relationships between executive subdomains and FAST outcomes in the healthy group (*N* = 59), expressed by standardized Beta coefficients.

**Executive subdomains**	**Psychosocial outcomes**						
	**FAST total score**	**Autonomy**	**Occupational functioning**	**Subjective cognition**	**Leisure time**	**Financial issues**	**Interpersonal relationships**
BCST Perseverative errors	0.109 (0.352)	0.037 (0.774)	0.131 (0.279)	−0.145 (0.274)	0.013 (0.921)	0.001 (0.990)	0.137 (0.225)
TOL Total Moves	−0.105 (0.373)	−0.126 (0.335)	−0.103 (0.397)	0.062 (0.640)	−0.153 (0.238)	−0.047 (0.679)	−0.137 (0.262)
Stroop task Incongruency errors	0.178 (0.139)	0.207 (0.120)	0.217 (0.081)	−0.098 (0.424)	0.168 (0.201)	0.239 (0.043)^*^	0.273 (0.030)^*^

p-values are presented in parentheses.

* *Significant at p < 0.05*.

*Linear regression model adjusted for age, gender, and years of education*.

### Remitted depression group

Perseverative errors (i.e., updating) were negatively associated with FAST total score in the remitted group, indicating that greater errors was associated with lower dysfunction. In contrast, neither forward planning nor inhibition was related to FAST total score in the remitted group (Table 4). Demographic factors (i.e., age, education, sex) were not reliably associated with total FAST score, however HAM-D score was positively associated FAST total (β = 0.766, *p* < 0.001). Analysis of FAST subdomains in the remitted depression group indicated that perseverative errors was significantly associated with occupational functioning and subjective cognition, with greater errors corresponding with reduced dysfunction. In contrast, the effects of TOL total moves and Stroop task incongruency errors on FAST subdomains did not approach significance. Statistics for the remitted group are presented in Table 3.

**Table 3:** Domain specific relationships between executive subdomains and FAST outcomes in the remitted depression group (*N* = 52), expressed by standardized Beta coefficients.

**Executive subdomains**	**Psychosocial Outcomes**						
	**FAST total score**	**Autonomy**	**Occupational functioning**	**Subjective cognition**	**Leisure time**	**Financial issues**	**Interpersonal relationships**
BCST perseverative errors	−0.215 (0.013)^*^	−0.141 (0.237)	−0.326 (0.014)^*^	−0.273 (0.017)^*^	−0.186 (0.171)	0.199 (0.184)	−0.028 (0.799)
TOL total moves	−0.022 (0.809)	0.108 (0.396)	−0.070 (0.608)	−0.069 (0.559)	−0.086 (0.552)	−0.045 (0.778)	0.133 (0.255)
Stroop task incongruency errors	0.027 (0.744)	−0.052 (0.654)	0.172 (0.175)	−0.006 (0.955)	0.078 (0.553)	−0.042 (0.774)	−0.045 (0.673)

p-values are presented in parentheses.

* *Significant at p < 0.05*.

*Linear regression model adjusted for HAM-D score, age, gender, and years of education*.

### Current depression group

The analysis of FAST total score in the currently depressed group indicated a significant relationship with TOL total moves (i.e., forward planning), such that poorer performance in the TOL task was associated with poor overall psychosocial functioning. In contrast, other executive subdomains were not independently associated with FAST total score. Demographic factors in the Current Depression group did not significantly affect overall functioning (i.e., total FAST score), but HAM-D score was positively associated with FAST total (β = 0.602, *p* = 0.006). FAST subdomain analyses revealed that TOL total moves was positively associated with autonomy, subjective cognition, leisure time, and interpersonal relationships, with poor forward planning associated with functional issues. BCST perseverative errors were negatively associated with dysfunction subjective cognition, albeit this effect was marginally significant. No effects of Stroop incongruency errors (i.e., inhibition) on FAST subdomains approached significance. Table 4 illustrates the relationships between executive subdomains and FAST outcomes in the current depression group.

**Table 4:** Domain specific relationships between executive subdomains and FAST outcomes in the current depression group (*N* = 31), expressed by standardized Beta coefficients.

**Executive subdomains**	**Psychosocial Outcomes**						
	**FAST total Score**	**Autonomy**	**Occupational functioning**	**Subjective cognition**	**Leisure time**	**Financial issues**	**Interpersonal relationships**
BCST perseverative errors	−0.218 (0.359)	−0.063 (0.783)	−0.417 (0.096)	−0.465 (0.075)^~^	−0.210 (0.403)	−0.161 (0.567)	0.147 (0.553)
TOL total moves	0.451 (0.012)^*^	0.420 (0.015)^*^	0.239 (0.174)	0.388 (0.039)^*^	0.427 (0.022)^*^	0.261 (0.198)	0.437 (0.019)^*^
Stroop task incongruency errors	−0.193 (0.442)	0.068 (0.777)	−0.359 (0.171)	−0.219 (0.415)	−0.218 (0.411)	0.188 (0.527)	−0.057 (0.825)

p-values are presented in parentheses.

~ Marginal,

* *Significant at p < 0.05*.

*Linear regression model adjusted for HAM-D score, age, gender, and years of education*.

## Discussion

The results suggest a dissociable impact of executive subdomains on functional outcomes. While the total participant sample did not show an effect of executive subdomains on psychosocial outcomes, these relationships became apparent in analysis of clinical subgroups (i.e., healthy, remitted depression, current depression). Specifically, in the currently depressed group deficits in forward planning (i.e., TOL total moves) were positively associated with overall psychosocial dysfunction, as well as specific functional issues in autonomy, subjective cognition, leisure time, and interpersonal relationships. In the remitted group, cognitive updating (i.e., BCST perseverative errors) was *negatively* associated with psychosocial dysfunction overall, as well as occupational functioning and subjective cognition. In contrast to the depressed and remitted groups, no executive subdomain was related to overall psychosocial functioning in the healthy group. However poor inhibition (i.e., Stroop task incongruency errors) were associated with financial and interpersonal difficulties in healthy individuals. These findings shed new light on the domain-specific relationships between executive cognition and psychosocial functioning in MDD. Specifically, it is suggested poor cognitive ability to plan future behavior may be an independent cause of psychosocial impairment during the acute stage of MDD. In contrast, over-sensitivity to incoming information (i.e., cognitive updating) may be detrimental to functional recovery during the remitted stage.

It is noteworthy that executive subdomains were differentially associated with psychosocial outcomes across in the remitted and currently depressed groups. Specifically, forward planning represented as the primary domain in currently depression, and cognitive updating as primary in remitted depression. The discrepant impact of forward planning and updating in these clinical subgroups may explain some of the inconsistency in the reported association between executive deficits and functional outcomes (5, 7). It is possible that studies of acute MDD, which employ measures primary reliant forward planning (e.g., the TOL task), may be more likely to identify a relationship between executive and functional ability than if other measures of executive functioning are used. In addition, studies which combine remitted and euthymic MDD patients into a single group (i.e., lifetime MDD) may report discrepant outcomes contingent upon which measure of executive functioning is employed (7).

A pertinent question raised by our data is why deficits in forward planning, rather than inhibition or updating, were independently associated with psychosocial disability in currently depressed patients. These findings suggest that ability to plan future behavior is more central to adaptive daily life than is capacity to inhibit unwanted responses, or ability to update one's perspective with new information. A plausible explanation is that forward planning enables depressed patients to anticipate and prioritize crucial functional responsibilities. For example, anticipating that social contact and interpersonal support are important in times of emotional hardship may enable one to maintain key relationships in their time of greatest need. Conversely, without such forward planning social networks may be neglected, resulting in social isolation and exacerbation of psychosocial dysfunction (14). The present findings support this example, as greater forward planning corresponded with lower interpersonal dysfunction. The primacy of forward planning suggests that cognitive and psychosocial remediation programs may be improved by emphasizing this subdomain as a primary treatment target (13, 35).

The findings in the remitted group were unexpected, as deficits in cognitive updating were associated with reduced, rather than increased, psychosocial dysfunction overall, and in domains of occupational functioning and subjective cognition. These findings are divergent from the prevailing literature, which suggests that cognitive deficits are associated with poor psychosocial functioning (1, 4, 5). However, it should be noted that the wider literature pertains chiefly to currently depressed patients. As such, it is worth considering how executive abilities may contribute differentially during the remitted, as opposed to the euthymic, stage. It is possible, for instance, that deficits in cognitive updating reduce sensitivity to negative feedback, since negative information may not be incorporated as readily in those with cognitive updating deficits. Given that oversensitivity to negative feedback may be a trait marker for MDD (36), it stands to reason that any mechanism diminishing its effect could benefit psychosocial functioning in general. In any case, future investigations of psychosocial functioning in remitted depression should consider cognitive updating separately from other executive subdomains, as its potentially protective effect may obscure the negative influence of other cognitive domains on functional outcomes (7).

In the healthy group, no executive subdomains were associated with overall psychosocial functioning, however poor inhibition was related to interpersonal and financial issues. These results bear discussion, as the question is raised as to why executive performance was associated with overall functioning in the MDD groups, but not in the healthy control group. It is possible that the effect of executive subdomains on overall functioning in the healthy group was obscured by the contribution of age and years of education, which were positively associated with overall dysfunction in the healthy group, but were not related to overall functioning in the MDD groups. The independent contribution of inhibition to financial management and interpersonal relationships highlights the importance of monitoring and suppressing inappropriate spending and poor social behavior (32).

It should be acknowledged that a clinical interview was utilized in the current study to evaluate psychosocial functioning. Accordingly, our results indicate clinician's perception of patient dysfunction, and require further validation with objective or performance-based measures [e.g., the performance assessment of self-care skills (PASS)] (37). It is also noteworthy that the current analyses consider cognitive updating, inhibition and forward planning separate entities. In reality these executive subdomains overlap, as they draw upon a shared pool of limited capacity cognitive resources (22, 38). As a result, demonstrated performance in executive subdomains (e.g., TOL vs. BCST) is not entirely dissociable. Finally, due to the relatively modest sample size in the current study (*N* = 142), further validation is needed in larger clinical samples with equally well-characterized clinical subgroups (i.e., remitted depression, currently depressed, HCs).

In summary, the current findings indicate distinct relationships between executive subdomains and functional outcomes across clinical subgroups. Deficits in forward planning are associated with increased dysfunction in a number of functional outcomes in currently depressed, whereas decreased cognitive updating appears to be protective to functional outcomes in remitted patients. In contrast, overall functioning in healthy individuals is not associated with executive subdomains. Taken together, these findings highlight a complex and domain-specific relationship between of executive subdomains and functional outcomes. Further investigation of these relationships, as well as the clinical efficacy of their treatment in MDD, is warranted.

## Author contributions

BB coordinated and supervised the CoFaM-S study, from which the current results were derived. MK and BB mutually developed the hypotheses and conceptual outline of the present manuscript. MK analyzed data and wrote the manuscript, while BB edited and provided feedback during the writeup.

### Conflict of interest statement

BB received speaker/consultation fees from: AstraZeneca, Lundbeck, Pfizer, Takeda, Servier, Bristol Myers Squibb, Otsuka, and Janssen-Cilag. MK declares that the research was conducted in the absence of any commercial or financial relationships that could be construed as a potential conflict of interest.

## Footnotes

^1^Major Depression was considered the primary illness according to the results of the MINI 600, even in those patients indicating anxiety symptoms.

^2^Berg's card sorting test is a digital freely available variant of the original Wisconsin Card Sorting Test, which includes modified instructions suitable for the digital format.

^3^Statistics for the HC regression model with HAM-D scores included as a covariate are reported in Supplementary eTable 3.
