# Supplementary material for: Executive Subdomains Are Differentially Associated With Psychosocial Outcomes in Major Depressive Disorder
Source: Front Psychiatry. 2018 Jul 10;9:309. doi: 10.3389/fpsyt.2018.00309 (PMC6048277; doi:10.3389/fpsyt.2018.00309)
Supplement: Supplementary file 1 [file Table_1.DOCX]

| **Supplementary eTable 1** | | |
| --- | --- | --- |
| Executive subdomains and performance metrics by cognitive test. | | |
| Cognitive test | Performance metric | Executive subdomain |
| Stroop task | Incongruency errors | Inhibition |
| Tower of London | Total moves | Forward planning |
| Becks Card Sorting Test | Perseverative errors | Updating |
