# Supplementary material for: Executive Subdomains Are Differentially Associated With Psychosocial Outcomes in Major Depressive Disorder
Source: Front Psychiatry. 2018 Jul 10;9:309. doi: 10.3389/fpsyt.2018.00309 (PMC6048277; doi:10.3389/fpsyt.2018.00309)
Supplement: Supplementary file 2 [file Table_2.DOCX]

| **Supplementary eTable 2** | | | | | | | |  |  |  |
| --- | --- | --- | --- | --- | --- | --- | --- | --- | --- | --- |
| Mean FAST score, FAST subdomain score, and performance in executive subdomains. Standard deviations are shown in parentheses. | | | | | | | | | | |
|  | Psychosocial Outcomes | | | | | | | Executive Subdomains | | |
|  | FAST total Score | Autonomy | Occupational Functioning | Subjective Cognition | Leisure Time | Financial Issues | Interpersonal relationships | BCST Perseverative errors | TOL  Total Moves | Stroop task Incongruency errors |
| Current | 26.97 (16.71) | 4.03 (3.51) | 5.06 (4.33) | 7.16 (3.65) | 2  (1.95) | 1.16 (1.92) | 7.55 (5.36) | 18.77 (4.77) | 221.58 (45.99) | 4.16 (5.87) |
| Remitted | 11.72 (11.24) | 1.28 (2.40) | 2.62 (3.25) | 4.32 (3.26) | .79 (1.12) | .42  (.95) | 2.29 (3.73) | 16.38 (5.84) | 226.19 (44.39) | 4.36 (5.29) |
| Healthy | 8.37 (10.94) | .82  (1.92) | 1.90 (3.12) | 2.90 (2.95) | .60 (1.10) | .70  (1.39) | 1.45 (2.81) | 19.07 (8.18) | 226.13 (54.75) | 4.05 (5.22) |
|  | | | | | | | |  |  |  |
