# Supplementary material for: Executive Subdomains Are Differentially Associated With Psychosocial Outcomes in Major Depressive Disorder
Source: Front Psychiatry. 2018 Jul 10;9:309. doi: 10.3389/fpsyt.2018.00309 (PMC6048277; doi:10.3389/fpsyt.2018.00309)
Supplement: Supplementary file 3 [file Table_3.DOCX]

| **Supplementary eTable 3** | | | | | | | | | |
| --- | --- | --- | --- | --- | --- | --- | --- | --- | --- |
| Domain specific relationships between executive subdomains and FAST outcomes in the **healthy** group (*N*= 59), with HAM-D score included in the model. Beta coefficients are provided with associated *p* values in parentheses. | | | | | | | | | |
| Executive subdomains | Psychosocial Outcomes | | | | | | | | |
|  | FAST total Score | | Autonomy | Occupational Functioning | Subjective Cognition | Leisure Time | | Financial Issues | Interpersonal relationships |
| HAM-D Score | | .719  (<.001)** | .635 (<.001) ** | .566 (< .001) ** | .521 (<.001) ** | .515  (<.001) ** | | .541  (< .001)** | .711  (< .001)** |
| BCST Perseverative errors | | .012 (.867) | -.048 (.636) | .055 (.578) | .032(.752) | -.057 (.612) | | -.071 (.438) | .042  (.602) |
| TOL  Total Moves | | -.034 (.645) | -.063 (.541) | -.047 (.633) | .063 (.540) | -.102 (.365) | | .006 (.945) | -.066  (.412) |
| Stroop task Incongruency errors | | .103 (.171) | .141  (.178) | .159  (.116) | -.197 (.061) | .114 (.315) | | .182 (.055) | .199  (.018)* |
| *= Significant at *p*< .05, **= Significant at *p*< .001 Linear regression model adjusted for age, gender, and years of education. | | | | | | |  |  |  |
